# Predicting MYCN amplification in paediatric neuroblastoma: development and validation of a 18F-FDG PET/CT-based radiomics signature

**DOI:** 10.1186/s13244-023-01493-8

**Published:** 2023-11-24

**Authors:** Luo-Dan Qian, Shu-Xin Zhang, Si-Qi Li, Li-Juan Feng, Zi-Ang Zhou, Jun Liu, Ming-Yu Zhang, Ji-Gang Yang

**Affiliations:** Nuclear Medicine Department, Beijing Friendship Hospital, Capital Medical University, Beijing, 100050 China

**Keywords:** Neuroblastoma, 18F-FDG PET/CT, Radiogenomics, MYCN

## Abstract

**Objectives:**

To develop and validate an 18F-FDG PET/CT-based clinical-radiological-radiomics nomogram and evaluate its value in the diagnosis of MYCN amplification (MNA) in paediatric neuroblastoma (NB) patients.

**Methods:**

A total of 104 patients with NB were retrospectively included. We constructed a nomogram to predict MNA based on radiomics signatures, clinical and radiological features. The multivariable logistic regression and the least absolute shrinkage and selection operator (LASSO) were used for feature selection. Radiomics models are constructed using decision trees (DT), logistic regression (LR) and support vector machine (SVM) classifiers. A clinical-radiological (C-R) model was developed using clinical and radiological features. A clinical-radiological-radiomics (C-R-R) model was developed using the C-R model of the best radiomics model. The prediction performance was verified by receiver operating characteristic (ROC) curve analysis, calibration curve analysis and decision curve analysis (DCA) in the training and validation cohorts.

**Results:**

The present study showed that four radiomics signatures were significantly correlated with MNA. The SVM classifier was the best model of radiomics signature. The C-R-R model has the best discriminant ability to predict MNA, with AUCs of 0.860 (95% CI, 0.757–0.963) and 0.824 (95% CI, 0.657–0.992) in the training and validation cohorts, respectively. The calibration curve indicated that the C-R-R model has the goodness of fit and DCA confirms its clinical utility.

**Conclusion:**

Our research provides a non-invasive C-R-R model, which combines the radiomics signatures and clinical and radiological features based on 18F-FDGPET/CT images, shows excellent diagnostic performance in predicting MNA, and can provide useful biological information with stratified therapy.

**Critical relevance statement:**

Radiomic signatures of 18F-FDG-based PET/CT can predict MYCN amplification in neuroblastoma.

**Key points:**

• Radiomic signatures of 18F-FDG-based PET/CT can predict MYCN amplification in neuroblastoma.

• SF, LDH, necrosis and TLG are the independent risk factors of MYCN amplification.

• Clinical-radiological-radiomics model improved the predictive performance of MYCN amplification.

**Graphical Abstract:**

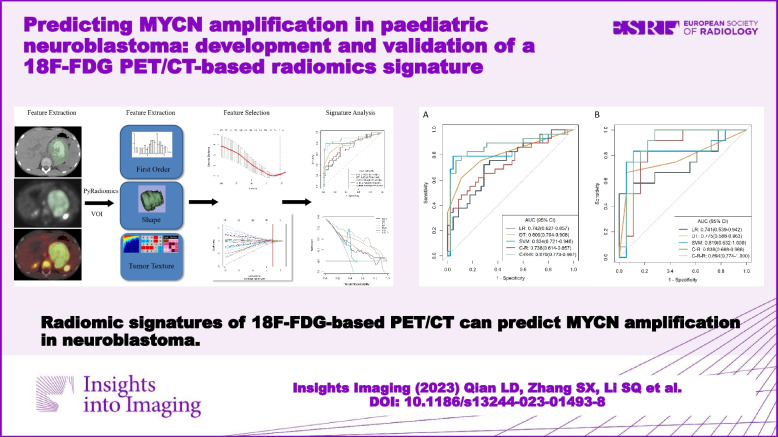

**Supplementary Information:**

The online version contains supplementary material available at 10.1186/s13244-023-01493-8.

## Introduction

Neuroblastoma (NB) is the most common extra cranial solid tumour of childhood. It can develop anywhere in the sympathetic nervous system, such as the adrenal gland or sympathetic ganglion, and it is characterised by significant tumour heterogeneity [1]. MYCN amplification (MNA) is one of the well-known prediction markers of poor outcome in NB [2] and is associated with 15–35% survival rate in high-risk patients, even in patients with otherwise favourable outcome profiles [3, 4]. As a result, detecting MNA is critical for the patients’ risk stratification. Traditional biopsy, on the other hand, may not always be accessible and result in a variety of complication [5]. Meanwhile, the availability of MYCN detection is hampered by the limited access to genetic assays by many institutions [6], hence the need to develop another non-invasive method to characterise MNA.

In recent years, the increasing application of radiomics in solid tumours has led to the emergence of radiogenomics. Radiogenomics is based on analysing high-dimensional quantitative signatures extracted from tumour regions of interest (ROIs) in radiological images [6] to identify and predict the expression of clinically significant molecular biomarkers of tumours [7]. Compared with histopathology and genetic testing methods, radiogenomics not only overcomes sampling bias and potential complications caused by biopsy but also is expected to provide more comprehensive and accurate information for predicting biomarkers [6].

Some studies attempted to predict MNA using a CT-based radiogenomics model [5, 8, 9]. However, these studies were limited by the lack of validation cohort, small sample size or lack of radiological features. In addition, although there was evidence that including clinical risk factors and radiological features could improve the diagnostic performance of the model, they had not been included in previous models [8.9]. Our previous studies have demonstrated the value of 18F-fluorodeoxyglucose positron emission tomography/computer tomography (18F-FDG PET/CT) based radiomics in the diagnosis of the mitosis-nuclear rupture index in paediatric NB and in predicting recurrence in high-risk patients [10, 11].

As a result, in this study, we developed a radiogenomic model based on the radiomics signature of 18F-FDGPET/CT before treatment to predict MNA paediatric patients. In addition, we present a visual radiogenomics nomogram that incorporates radiomics signatures, clinical factors, and radiological features.

## Materials and methods

### Patients

**Fig. 1:**
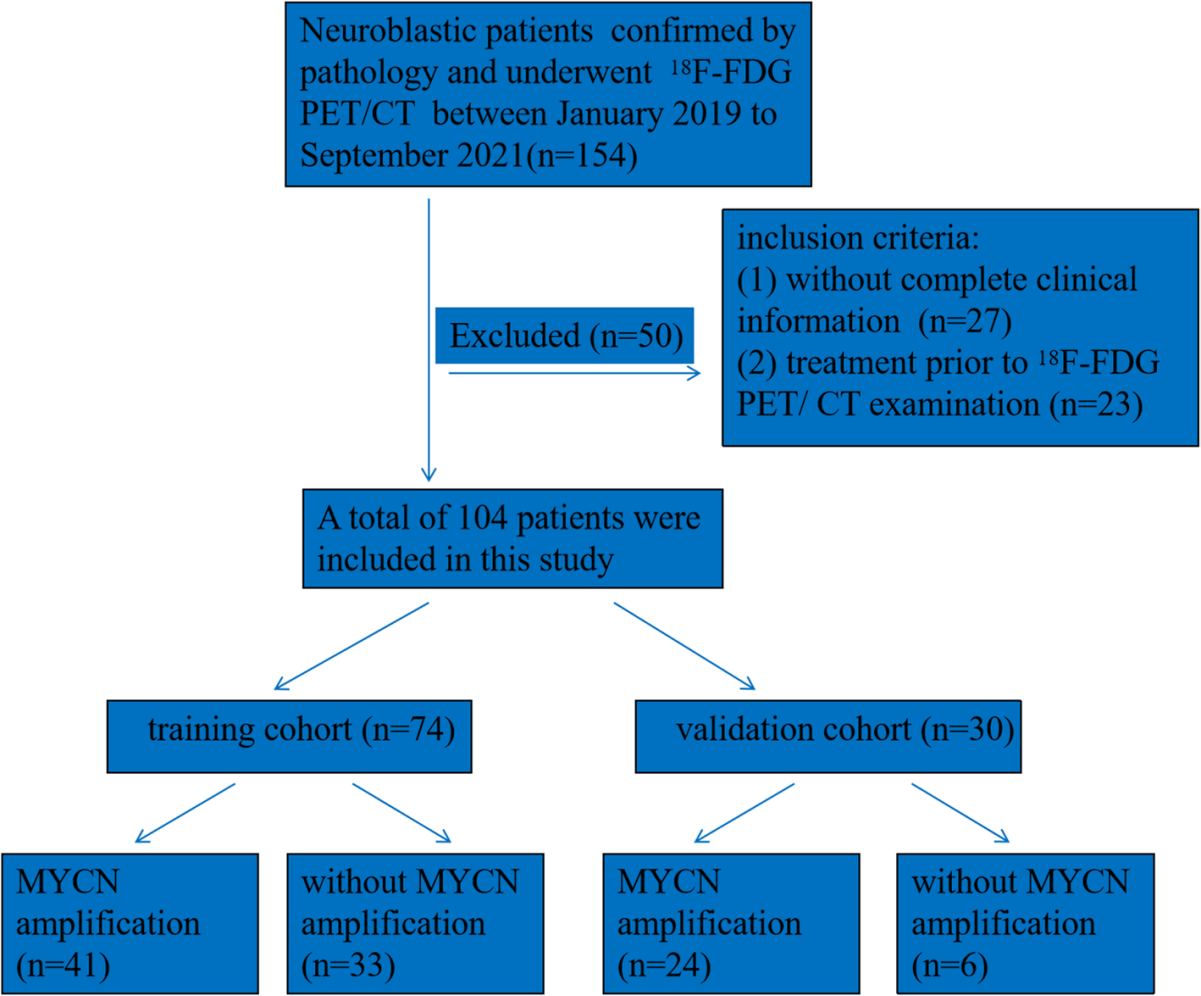
The flow chart for patient selection

Our institution’s institutional review board approved this retrospective study. From January 2019 to September 2021, we obtained the clinical data of 154 patients with NB confirmed by pathology and collected their preoperative 18F-FDG PET/CT images. The inclusion criteria were as follows: (1) pathologically confirmed NB; (2) ≤ 18 years at first diagnosis; (3) A 18F-FDG PET/TC scan was made before operation or biopsy (within 4 weeks) (4) without any radiotherapy, chemotherapy or surgical treatment received before the 18F-FDG PET/CT examination included or of interest; (5) complete clinical information (Laboratory examination and bone marrow biopsy results); (6) available MNA data. Subsequently, 50 patients were excluded, including 27 patients without complete clinical information, and 23 patients had the above treatment at first diagnosis. According to the result of biopsy or surgery, there were 65 patients with MNA and 39 patients without MYCN amplification (Wild). All patients were assigned to the training and validation cohorts at random in a 7:3 ratio. Figure 1 depicts the flow chart for patient selection.

The baseline data of each patient were obtained by reviewing the medical records and 18F-FDGPET/CT imaging. Clinical factors included age, gender, neuron-specific enolase (NSE), serum ferritin (SF), lactate dehydrogenase (LDH), vanillylmandelic acid (VMA) and homovanillic acid (HVA) level in a 24-h urine sample.

The radiological features (Table 1) of all patients were evaluated on a workstation (syngo.via, Siemens) by two nuclear medicine physicians with 5 and 10 years of experience in paediatric oncology diagnosis, respectively. But they were blinded to the clinical and histopathological diagnosis. In the event of a disagreement, a consensus was reached after further discussion. 18F-FDGPET/CT radiological features included International Neuroblastoma Risk Group Staging System (INRGSS), anatomical compartment, infiltration across the midline, calcification, and necrosis. Meanwhile, three conventional PET parameters were extracted from primary tumours (maximum standard uptake values (SUVmax), metabolic tumour volume (MTV), and total lesion glycolysis (TLG). Table 1 summarises the clinical factors and radiological features of the patients in the training and validation cohorts.

**Table 1:** Characteristics of patients with neuroblastoma in the training cohort and validation cohort

Characteristics	All Patients (*n* = 104)	Training cohort (*n* = 74)	Validation cohort (*n* = 30)	*p* value
**MYCN gene status**				0.263
Amplified	65 (62.5%)	41 (55.4%)	24 (80.0%)	
Not Amplified	39 (37.5%)	33 (44.6%)	6 (20.00%)	
**Clinical factors**				
Age at diagnosis				0.828
≥ 18 mos at diagnosis	80 (76.9%)	56 (75.7%)	24 (80.0%)	
< 18 mos at diagnosis	24 (23.1%)	18 (24.3%)	6 (20.0%)	
Gender				0.541
Female	62 (59.6%)	46 (62.2%)	16 (53.3%)	
Male	42 (40.4%)	28 (37.8%)	14 (46.7%)	
High NSE (> 16.3 ng/mL)	103 (99.0%)	73 (98.7%)	30 (100.0%)	1.000
Normal NSE(≤ 16.3 ng/mL)	1 (1.0%)	1 (1.3%)	0 (0.0%)	
High SF (> 115 ng/mL)	49 (47.1%)	32 (43.2%)	17 (56.7%)	0.305
Normal SF(≤ 115 ng/mL)	55 (52.9%)	42 (56.8%)	13 (43.3%)	
High LDH(> 662.5 U/L)	36 (34.6%)	24 (32.4%)	12 (40.0%)	0.612
Normal LDH(≤ 662.5 U/L)	68 (65.4%)	50 (67.6%)	18 (60.0%)	
High VMA (> 68.6 μmo/L)	70 (67.3%)	50 (67.6%)	20 (66.7%)	1.000
Normal VMA(≤ 68.6 μmo/L)	34 (32.7%)	24 (32.4%)	10 (33.3%)	
High HVA(> 40 μmol/L)	56 (53.8%)	36 (48.7%)	20 (66.7%)	0.146
Normal HVA(≤ 40 μmol/L)	48 (46.2%)	38 (51.3%)	10 (33.3%)	
**PET/CT radiological features**				
INRGSS				0.920
L1, L2, MS	39 (37.5%)	42 (56.8%)	16 (53.3%)	
M	65 (62.5%)	32 (43.2%)	14 (46.7%)	
Thoracic primary				1.000
Yes	13 (12.5%)	9 (12.2%)	4 (13.3%)	
No	91 (87.5%)	65 (87.8%)	26 (86.7%)	
Adrenal primary				0.705
Yes	73 (70.2%)	52 (70.3%)	21 (70.0%)	
No	31 (29.8%)	22 (29.7%)	9 (30.0%)	
Infiltrating across midline				0.473
Yes	48 (46.2%)	32 (43.2%)	16 (53.3%)	
No	56 (53.8%)	42 (56.8%)	14 (46.7%)	
Calcification				0.200
Yes	72 (69.2%)	48 (64.9%)	24 (80.0%)	
No	32 (30.8%)	26 (35.1%)	6 (20.0%)	
Necrosis				0.887
Yes	77 (74.0%)	54 (73.0%)	23 (76.7%)	
No	27 (26.0%)	20 (27.0%)	7 (23.3%)	
SUVmax (Median ± IQR)	4.600 (3.8, 6.200)	4.500 (3.625, 6.175)	4.750 (4.225, 6.150)	0.482
MTV (mL) (Median ± IQR)	116.8 (40.2, 208.5)	221.650 (73.700, 415.625)	322.750 (137.050, 632.825)	0.113
TLG (Median ± IQR)	253.5 (81.6, 486.2)	103.750 (39.475, 163.450)	136.750 (48.600, 291.000)	0.149

*NSE* Neuron-specific enolase, *SF* Serum ferritin, *LDH* Lactate dehydrogenase, *VMA* VAnillylmandelic acid, *HVA* Vanillylmandelic acidand, *INRGSS* International Neuroblastoma Risk Group Staging System, *SUVmax* Maximum standard uptake values, *MTV* Metabolic tumour volume, *TLG* Total lesion glycolysis, *L1* Localised tumour not involving vital structures as defined by the list of image-defined risk factors and confined to one body compart men, *L2* Locoregional tumour with presence of one or more image-defined risk factors, *M* Distant metastatic disease (except stage MS), *MS* Metastatic disease in children younger than 18 months with metastases confined to skin, liver, and/or bone marrow

### Analysis of the MYCN gene status

MNA was determined using FISH from paraffin-embedded tissue obtained by biopsy or surgery at the time of initial diagnosis, as previously published [12]. MNA was defined as a > fourfold increase in signals, according to the European Neuroblastoma Quality Assessment group [13, 14].

### PET/CT image acquisition

All patients were performed with a full body (from apex to toe) 18F-FDGPET/CT scanner (Biograph mCT-64 PET/CT; Siemens) [15]. They were asked to fast for at least 6 h and cut back on high-intensity exercise for at least 24 h before the injection. 18F-FDG (provided by Beijing Atomic High-tech Company) were injected intravenously 40–60 min before PET/CT scan. First, anatomical reference and attenuation correction were performed using low-dose CT scans, corrected with 120 kV tube voltage and auto-modulated tube current. CT image parameters are as follows: resolution 0.586 mm × 0.586 mm, slice thickness 2 mm, matrix size 512 × 512. PET scan was performed immediately after whole-body CT scan for 2 min per bed. The ordered subset expectation maximisation (OSEM) time-of-flight (TOF) algorithm was used to reconstruct PET images. PET image parameters are as follows: resolution 4.07 mm × 4.07 mm, slicer thickness 3 mm, matrix size 200 × 200.

### Radiomics signature selection

Tumour segmentation and feature extraction were done as follows: to ensure the quality of the extracted radiomics features, 3D Slicer (version 4.10.2, funded by the National Institutes of Health) was used for medical image registration. The primary tumour delineation was performed using fixed SUV threshold method. According to the result of previous studies, 40% of SUVmax is set as the threshold for the images [16–18]. In this method, 3-D contours are drawn around voxels equal to or greater than 40% SUVmax. For the volume of interest (VOI) containing more than one cluster, the cluster which has maximum uptake intensity and volume is selected. A manual verification after automatic segmentation was performed; special attention was paid to tumour located near the urinary bladder due to intense physiological urinary tracer activity. If the VOI was found to be incorrect, additional manual adjustments were required. The VOIs included the lesion’s calcification and necrosis areas [19]. To minimise between-observer differences [20], each VOI was confirmed by two children’s nuclear medicine doctors (Q.L.D. and W.W.). For each precisely segmented VOI, the radiomics signature in the VOI was automatically extracted using radiomics in the open-source Python package (https://pyradiomics.readthedocs.io/en/Latest/). In each VOI, 1720 radiomics signatures were extracted from PET and CT images. These signatures include the following: (1) first-order features, (2) shape features, (3) and texture signatures (including grey-level co-occurrence matrix signatures (GLCM); grey-level dependence matrix signatures (GLDM); grey-level size zone matrix signatures (GLSZM); grey-level run-length matrix signatures (GLRLM); and neighbouring grey-tone difference matrix signatures (NGTDM)); we used Laplacian of Gaussian (LoG, sigma= 1, 3, 5, 7) and wavelet filtering to extract texture features.

Three months later, 40 patients were randomly selected from the training cohort to evaluate the reproducibility and robustness of the signature extraction process, and the nuclear medicine doctors (Q.L.D.) divided the data again and constructed a re-divided cohort. A value greater than 0.80 indicates good agreement when calculating intra/interclass correlation coefficients (ICCs).

**Fig. 2:**
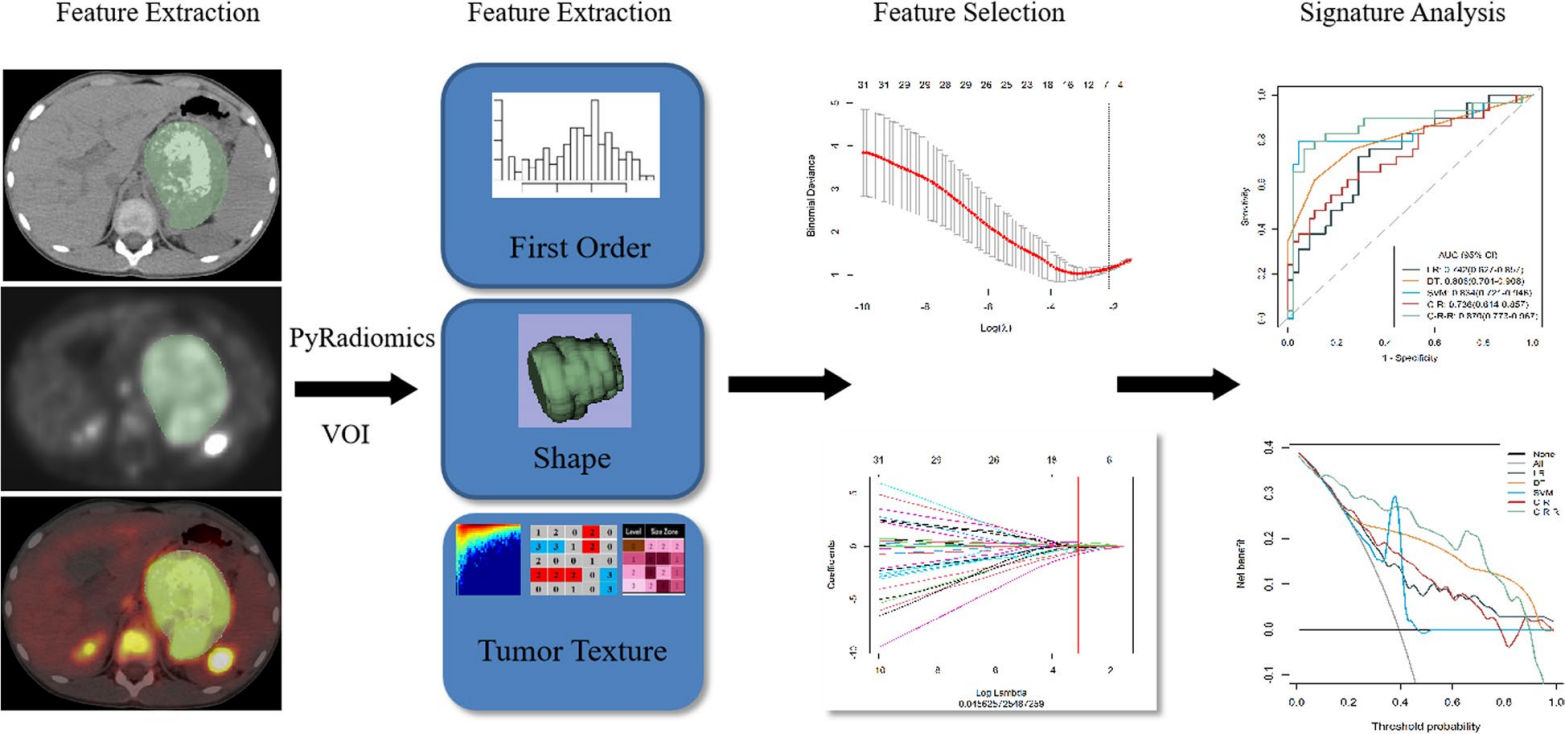
Radiomics signature workflow

Signature selection was done as follows: (1) use the *z*-score method to standardise all radiomics signatures in the training cohort; (2) Mann-Whitney *U* test retain signatures with *p* values less than 0.05; (3) Spearman correlation analysis and remove signatures with a correlation coefficient less than 0.9; (4) to find the most relevant predictive signatures, the least absolute shrinkage selection operator (LASSO) was used. Radiomics features underwent a multi-step selection process to overcome the limitations of traditional logistic regression methods, namely overfitting and multicollinearity problems in modelling high-dimensional radiomics signatures. The workflow was presented in Fig. 2.

Constructing radiomics signatures was done as follows: after removing the redundant signatures, we feed the last set of radiomics signatures into the classifier to create a radiomics feature, which was used for biological assessment. In this study, we evaluated three classifiers: logistic regression (LR), decision tree (DT) and support vector machine (SVM). All classifiers choose the best performing model by using 10-fold cross validation in training cohort. To evaluate the performance of different radiomics models, the area under the curve (AUC), sensitivity, specificity, and accuracy were calculated using the receiver operating characteristic (ROC) curve, and the best model was the selected radiomics model with the highest AUC.

### Establishment and evaluation of the models

The clinical and radiological features were chosen primarily for their association with the MNA [21]. First, univariate analysis was used to identify clinical and radiological features that differed significantly from the MNA in the training cohort; then, multiple logistic regression analysis was used to identify the most relevant variables. Following multivariate analysis, clinical and radiological features independently related to MNA were used to develop a clinical-radiology (C-R) model. The radiomics model with the highest AUC outputs probability values for everyone, which are combined with clinical-radiological parameters to construct a multivariable logistic regression model (clinical-radiological-radiomics; C-R-R) and calculate the diagnostic efficiency of the model. The calibration curve and the Hosmer-Lemeshow test [22] were used to assess the model’s goodness of fit. To evaluate the clinical effectiveness of the model, a decision curve analysis (DCA) was used for the training and validation cohorts to calculate the net benefits under the threshold probability.

### Statistical analysis

Statistical analyses were performed using R (version 4.1.0, Statistical Calculation Basics). A two-sided *p* value of less than 0.05 was considered statistically significant. Differences in all clinical features between two groups were assessed by independent samples *t* test, Mann-Whitney *U* test, and chi-squared or Fisher’s exact tests, as appropriate. DeLong test was used to compare the differences in AUC values between models. Accuracy, specificity, and sensitivity were calculated based on the cut-off value of the maximum Youden index.

## Results

### Clinical and radiological features of patients

The patients were randomly divided into a training and validation cohort in the ratio of 7:3. There was no statistically significant difference in MNA rates and clinical and imaging features between the two cohorts (*p* > 0.05). The rate of MNA in total, training, and validation groups was 62.5% (65/104), 55.4% (41/74), and 80.0% (24/30), respectively (Table 1).

Ten significant clinical and radiological features were identified by univariate regression analysis (Table 2). Multivariate logistic analysis revealed that SF (odds ratio [OR], 1.484; 95% confidence interval [CI], 1.063–2.105; *p* = 0.048), LDH (OR, 5.825; 95% CI, 2.015,18.489; *p* = 0.002), necrosis (OR, 2.206; 95% CI, 0.747,7.216; *p* = 0.167), and TLG (OR, 1.001; 95% CI, 1.000, 1.002; *p* = 0.156) were correlated independently with the MNA status (Table 2).

**Table 2:** Univariate and multivariate logistic regression analysis of risk factors associated with MYCN amplified in the training cohort

Characteristics	Univariate		Multivariate	
	OR (95% CI)	*p* value	OR (95% CI)	*p* value
Clinical factors				
Age at diagnosis (years, ≥ 18 mos)	0.711 (0.279, 1.828)	0.008	-	-
Gender (Male)	2.081 (0.928, 4.736)	0.045	-	-
NSE (ng/mL, high)	1.004 (1.002, 1.005)	0.020	-	-
SF (ng/mL, high)	1.308 (0.591, 2.912)	< 0.001	1.484 (1.063–2.105)	0.048
LDH (U/L, high)	5.843 (2.452, 14.698)	< 0.001	5.825 (2.015, 18.489)	0.002
VMA (μmol/L, high)	0.897 (0.387, 2.106)	0.800	-	-
HVA (μmol/L, high)	1.361 (0.614, 3.059)	0.449	-	-
INRGSS (M)	1.770 (0.615, 5.489)	0.294	-	-
Thoracic primary (Yes)	0.664 (0.163, 1.248)	0.171	-	-
Adrenal primary (Yes)	1.535 (0.640, 3.870)	0.691	-	-
Infiltrating across midline (Yes)	3.325 (1.262, 9.337)	0.015	-	-
Calcification (Yes)	3.680 (1.362, 10.570)	0.064	-	-
Necrosis (Yes)	1.024 (0.353, 2.916)	0.010	2.206 (0.747, 7.216)	0.167
SUVmax (Median ± IQR)	1.123 (0.969, 1.301)	0.070	-	-
MTV (Median ± IQR, mL)	1.001 (1.000, 1.002)	0.006	-	-
TLG (Median ± IQR)	1.001 (0.998, 1.004)	< 0.001	1.001 (1.000, 1.002)	0.156
Rad score	1.338 (0.342, 5.233)	< 0.001	13.515 (3.364, 36.802)	< 0.001

*CI* Confidence interval, *OR* Odds ratio, *NSE* Neuron-specific enolase, *SF* Serum ferritin, *LDH* lactate dehydrogenase, *VMA* Vanillylmandelic acid, *HVA* Vanillylmandelic acidand, *INRGSS* International Neuroblastoma Risk Group Staging System, *M* distant metastatic disease (except stage MS), *MTV* Metabolic tumour volume, *TLG* Total lesion glycolysis

### Radiomics feature selection and signature construction

**Fig. 3:**
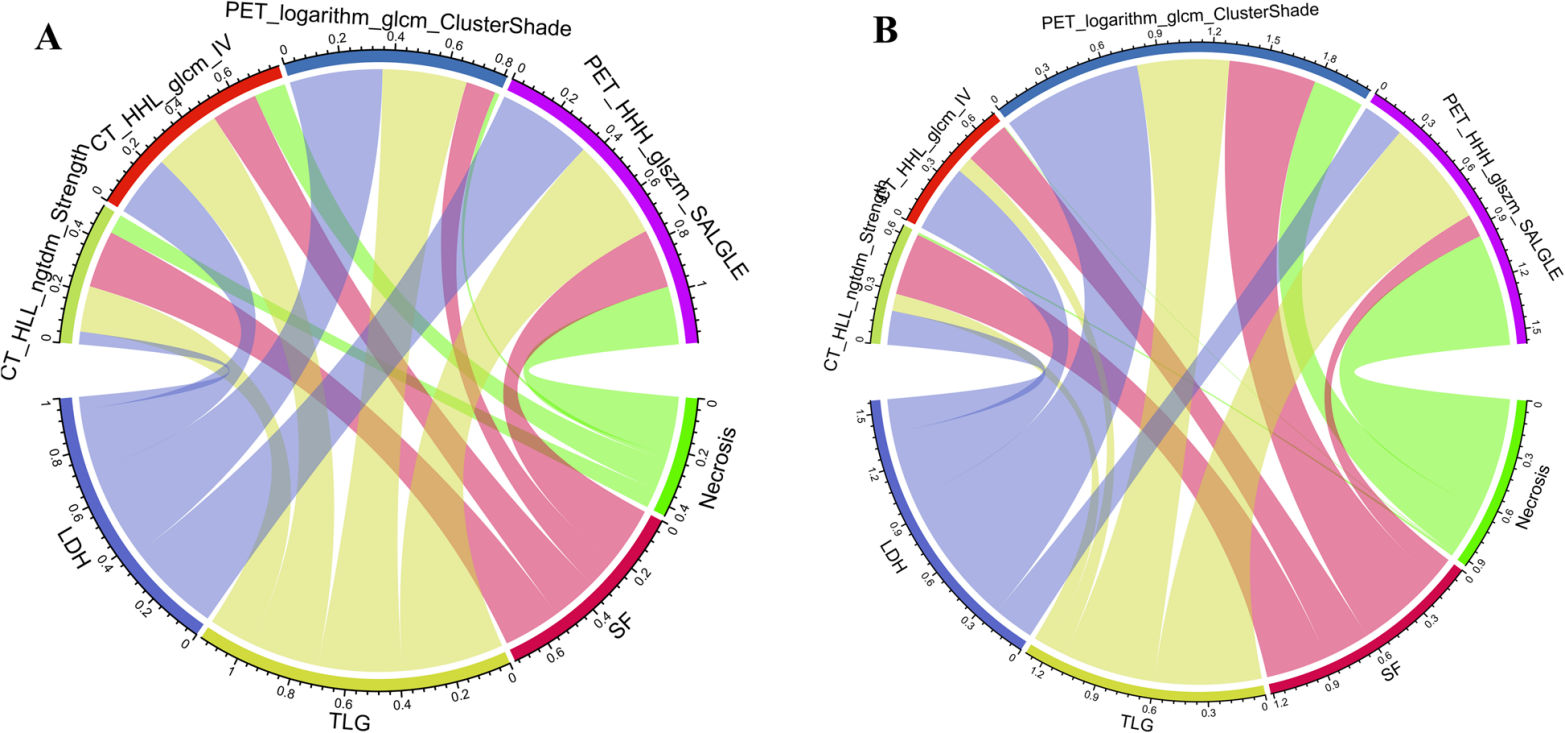
Chord diagram of the correlation between two clinical variable and two radiological and four radiomics features. Correlation analysis between selected radiomics features and clinical and radiological features in the training (**A**) and validation (**B**) cohorts

After assessing the robustness, 928 signatures were retained for model building, with ICC > 0.8. Two hundred and seventeen signatures were identified as independent after Mann-Whitney *U* test and correlation analyses. Finally, four radiomics signatures (2 PET and 2 CT radiomics signatures) with non-zero coefficients were selected after LASSO regression. The four radiomic signatures mentioned above were used in the training cohort to build three different radiomics models for predicting MNA, including LR, DT, and SVM classifiers. Figure 3 depicts the correlation analysis of two clinical factors and two radiological features with four radiomics signatures.

### Model comparisons

**Fig. 4:**
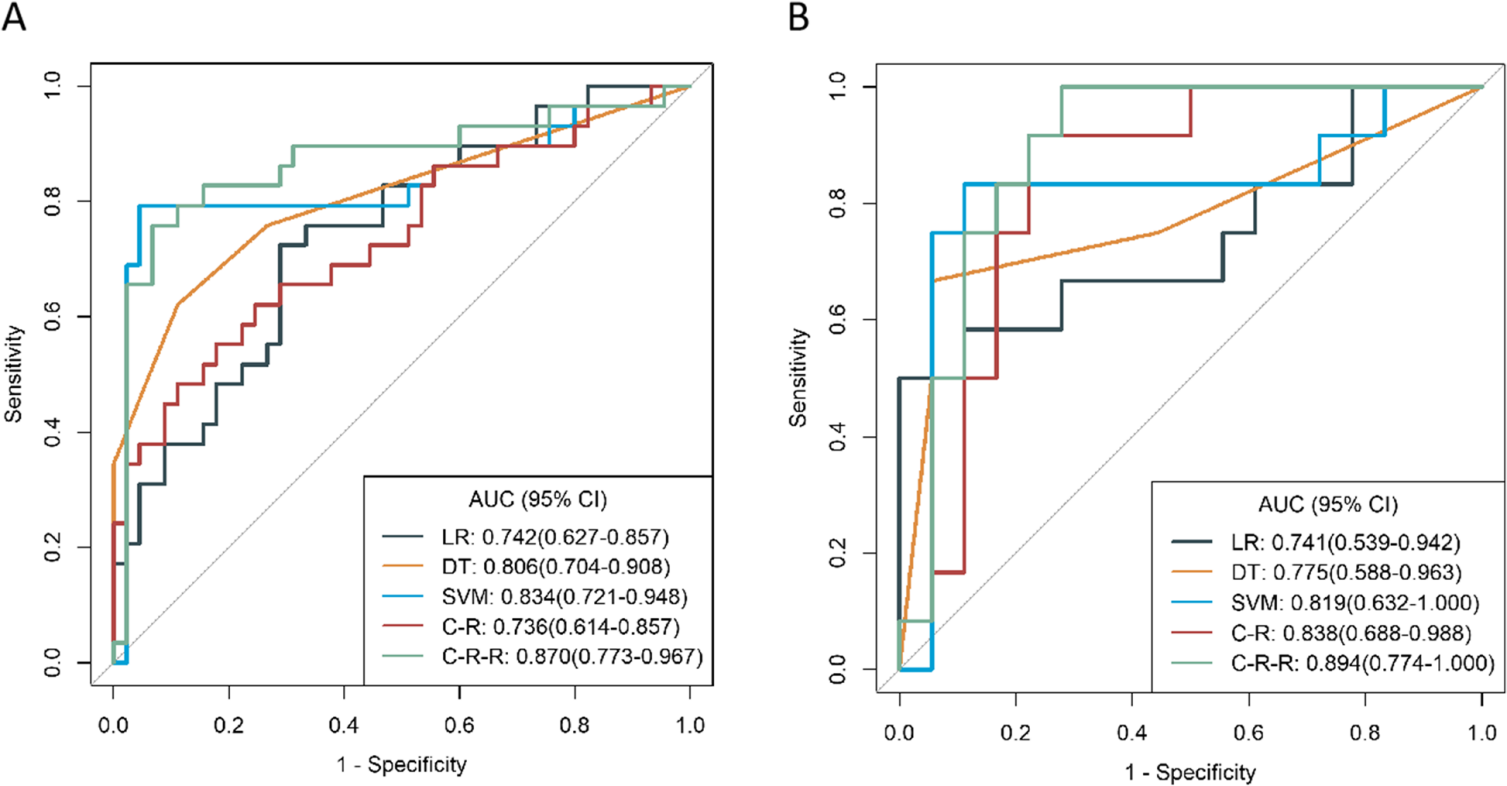
**A** Receiver operating characteristic (ROC) curves for the LR, DT, SVM classifiers, C-R model and C-R-R model in the training cohort. **B** ROC curves for the LR, DT, SVM classifiers, C-R model and C-R-R in the validation cohort. LR, logistic regression; DT, decision tree; SVM, support vector machine, C-R, clinical-radiological; C-R-R, clinical-radiological-radiomics

The diagnostic performance of the three radiomics and C-R and C-R-R models were shown in Table 3 and Fig. 4. In training and validation cohorts, the AUC values were 0.742 (95% CI, 0.627–0.857) and 0.741 (95% CI, 0.539–0.942) for LR and 0.806 (95% CI, 0.704–0.908) and 0.775 (95% CI, 0.588–0.963) for DT, respectively. The AUC values for the SVM in both cohorts were 0.834 (95% CI, 0.721–0.948) and 0.819 (95% CI, 0.632–1.000), respectively, which were higher than LR and DT classifiers. The AUC values of the C-R model in both cohorts were 0.672 (95% CI, 0.542–0.803) and 0.681 (95% CI, 0.468–0.893), respectively.

**Table 3:** The diagnostic performance of the different models for the MYCN amplification in the training and validation cohorts

	AUC (95%CI)	Accuracy (95%CI)	Sensitivity (95%CI)	Specificity (95%CI)
**Training cohort**				
LR	0.742 (0.627–0.857)	0.716 (0.599–0.815)	0.724 (0.379–0.897)	0.711 (0.400–0.844)
DT	0.806 (0.704–0.908)	0.784 (0.673–0.871)	0.621 (0.420–0.813)	0.889 (0.692–0.967)
SVM	0.834 (0.721–0.948)	0.892 (0.798–0.952)	0.793 (0.000–0.897)	0.956 (0.222–1.000)
C-R	0.672 (0.542–0.803)	0.716 (0.599–0.815)	0.345 (0.137–0.517)	0.956 (0.689–1.000)
C-R-R	0.860 (0.757–0.963)	0.878 (0.782–0.943)	0.793 (0.474–0.931)	0.933 (0.333–1.000)
**Validation cohort**				
LR	0.741 (0.539–0.942)	0.533 (0.343–0.717)	0.750 (0.500–1.000)	0.389 (0.165–1.000)
DT	0.775 (0.588–0.963)	0.833 (0.653–0.944)	0.667 (0.139–0.917)	0.944 (0.355–1.000)
SVM	0.819 (0.632–1.000)	0.867 (0.693–0.962)	0.833 (0.000–1.000)	0.889 (0.110–1.000)
C-R	0.681 (0.468–0.893)	0.633 (0.439–0.801)	0.500 (0.250–1.000)	0.722 (0.500–1.000)
C-R-R	0.824 (0.657–0.992)	0.867 (0.693–0.962)	0.750 (0.000–1.000)	0.944 (0.333–1.000)

*AUC* Area under the curve, *CI* Confidence interval, *C-R* Clinical-radiological, *C-R-R* clinical-radiological-radiomics, *DT* Decision tree; *LR* Logistic regression; *SVM* Support vector machine

**Fig. 5:**
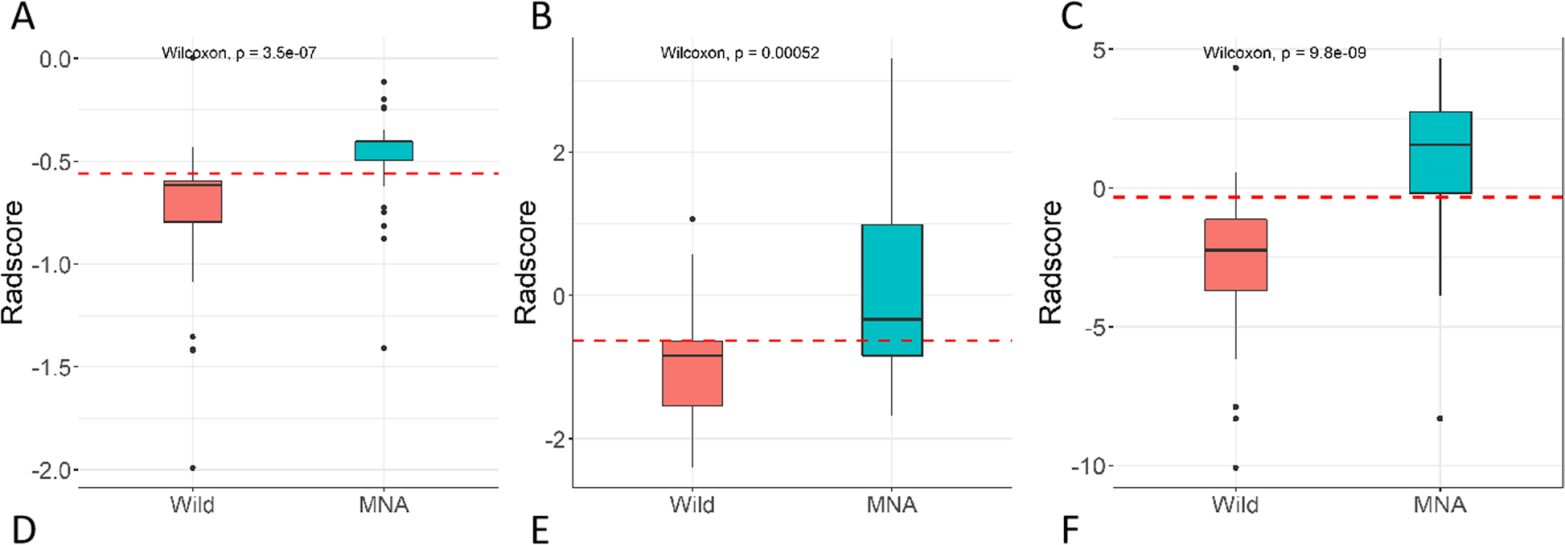
Boxplot showing the comparison of radiomics scores from the SVM classifier (**A**), C-R model (**B**), and C-R-R model (**C**) in the training. C-R, clinical-radiological; C-R-R, clinical-radiological-radiomics; SVM, support vector machine

The C-R-R model developed by combining the SVM and the C-R model obtained higher AUC values in the training and validation cohorts: 0.860 (95% CI, 0.757–0.963) and 0.824 (95% CI, 0.657–0.992), respectively. In addition, the C-R-R model had the best discrimination ability in both cohorts, with sensitivities of 0.793 (95% CI, 0.474–0.931) and 0.750 (95% CI, 0.000–1.000) and specificities of 0.933 (95% CI, 0.333–1.000) and 0.944 (95% CI, 0.333–1.000). In the training cohort, the SVM classifier and C-R model and C-R-R model scored significantly higher in the MNA group than the wild group. This result was confirmed in the validation cohort (Fig. 5).

Table A1 shows the results of the Delong tests performed on the different models. Based on these results, the C-R-R model significantly outperformed the SVM classifier (AUC, 0.834; *p* = 0.005), the LR classifier (AUC, 0.742; *p* < 0.001), the DT classifier (AUC, 0.801; *p* < 0.001) and the C-R model (AUC, 0.672; *p* < 0.001) in the training cohort. Again, in the validation cohort, the C-R-R model outperformed the C-R model and was significantly different from the C-R model (*p* = 0.007), but not from the other three classifiers (*p* > 0.05).

In short, the C-R-R model had high predictive value for MNA. Compared with other classifiers and C-R models, C-R-R model has higher AUC value, accuracy, and sensitivity in predicting MNA, and SVM classifier is superior to LR and DT classifier and C-R model.

### Evaluating model performance

**Fig. 6:**
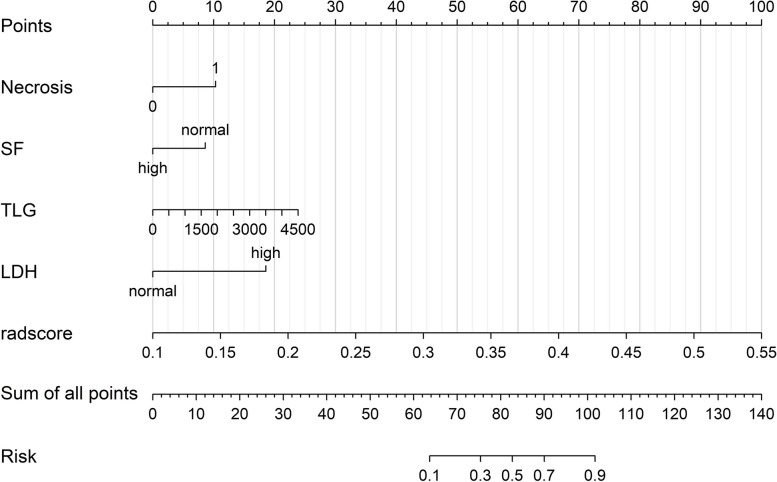
The nomogram developed based on the C-R-R model, which incorporated the Necrosis, SF, TLG, LDH and Rad score. It is used to non-invasively predict MYCN amplification in paediatric neuroblastoma patients. C-R-R, clinical-radiological-radiomics; SF, serum ferritin; TLG, total lesion glycolysis; LDH, lactate dehydrogenase

Based on the radiomics model with the highest AUC value, two clinical factors and two imaging features were combined to create a nomogram. Of the five models, the C-R-R model had the best discrimination ability (Figs. 4 and 6).

**Fig. 7:**
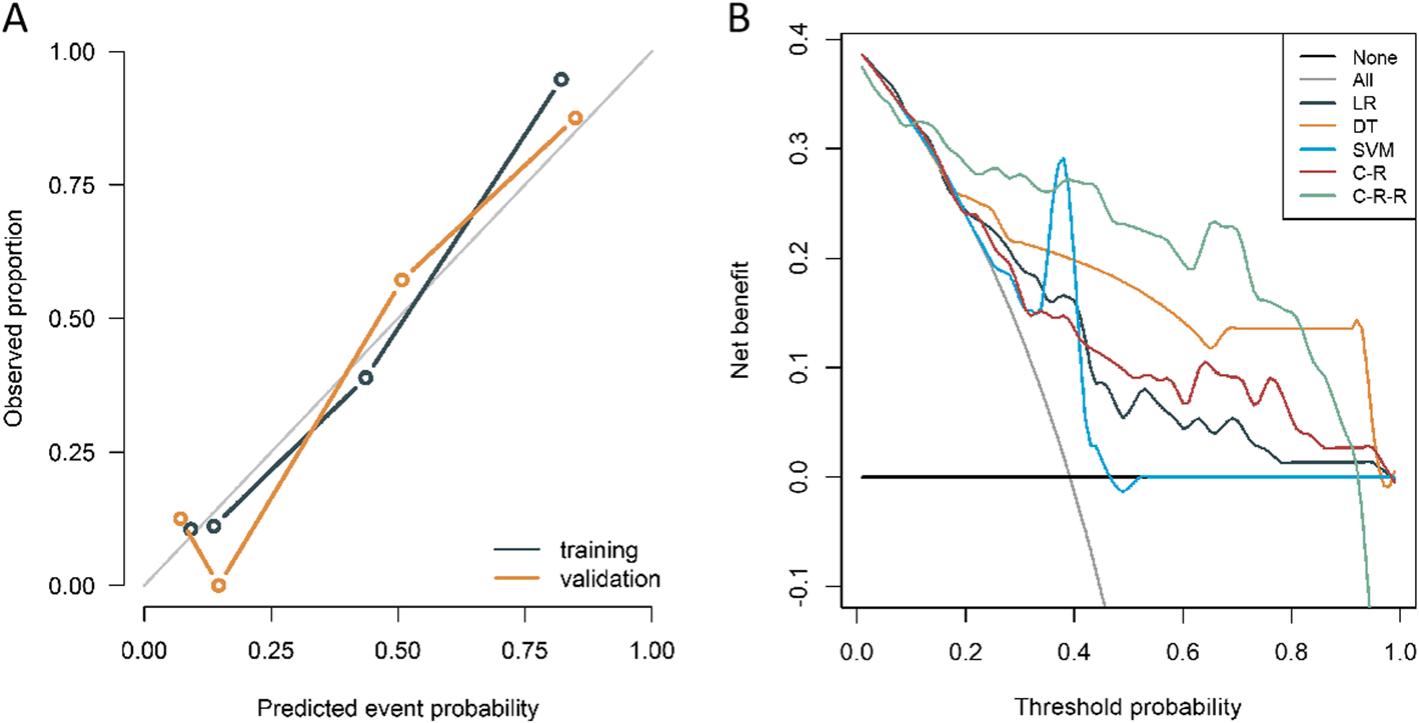
**A** Calibration curves of the C-R-R model in the training and validation cohorts. **B** Decision curve analysis (DCA) for the LR, DT, SVM classifiers, C-R model and C-R-R model in the training cohorts. **C** DCA for the LR, DT, SVM classifiers, C-R model and C-R-R model in the validation cohort. LR, logistic regression; DT, decision tree; SVM, support vector machine, C-R, clinical-radiological; C-R-R, clinical-radiological-radiomics

The calibration curve showed that the probability of the C-R-R model predicting MNA matched the actual probability well, while the Hosmer-Lemeshow test yielded an insignificant *p*-value, indicating no deviation from perfect fit (Fig. 7a). The threshold probability of the C-R-R model was 10–90%, and its area under the decision curve was greater than that of the other models in the training and validation cohorts, indicating that the model had optimal clinical utility (Fig. 7b).

## Discussion

In this study, we used a relatively large data set to build a C-R-R model based on the combination of an 18F-FDG PET/CT radiomics signature with clinical and radiological features to predict MNA in paediatric NB. We calculated the diagnostic performance of radiomic signatures using three classifiers (LR, DT, and SVM), and the SVM classifier with the highest AUC value was chosen. In the training and validation cohorts, the AUC values of the C-R-R model were higher, which were 0.860 and 0.824, respectively. Therefore, this model may help to determine the MNA of paediatric NB and guide personalised stratified treatment.

Our study found that age at initial diagnosis, gender and NSE, SF and LDH levels were significantly associated with MNA: male patients older than 18 months and with higher levels of NSE, SF and LDH were more likely to have MNA, which was consistent with that in previous studies [23, 24]. Besides, our findings showed that primary adrenal tumours were more frequently associated with MNA (OR, 1.535; 95% CI, 0.640,3.870; *p* = 0.691); in contrast, the number of patients with a primary thoracic tumour decreased (OR, 0.664; 95% CI, 0.163,1.248; *p* = 0.171), but no statistical significance was found. Five radiological features were significantly related to the MNA (*P* < 0.05). For example, necrosis, lesions infiltrating across the midline, higher TLG, SUVmax, and MTV values were more common in patients with MNA, which was consistent with previous studies [25, 26]. In multivariate logistic regression analysis, SF, LDH, necrosis and TLG were independently correlated with MNA (*P* < 0.05**)**.

To construct the radiomics signature, we screened 1720 candidate features for 4 independent features that were highly correlated with MNA and were stable in the validation cohort. These signatures were closely related to radiological features. For example, SF and LDH were significantly correlated with signatures such as CT_HLL_ngtdm_Strength, CT_HHL_glcm_IV and PET_logarithm_GLCM_ClusterShade, necrosis was significantly correlated with signatures such as PET_HHH_glszm_SALGLE and CT_HHL_glcm_IV and TLG was significantly correlated with signatures such as PET_HHH_glszm_SALGLE and PET_logarithm_GLCM_ClusterShade. The correlation between these features shows that although the texture-based signatures are invisible to the naked eye, the specific combination of several texture signatures can be explained by some radiological features to a certain extent.

To our knowledge, this is the first report on prediction of the MNA using a relatively large data set based on 18F-FDG PET/CT radiomics signature combined with clinical and radiological features. Meanwhile, we evaluated the performance of radiomic model using three different classifiers. Finally, the SVM classifier with the highest diagnostic performance was selected. Wu H et al. [5] reported that a model based on a three-phase CT radiomics signature could be used to predict MNA in paediatric NB with an AUC of 0.82 in the training cohort and only 0.70 in the validation cohort; however, the study did not include clinical factors. Chen X et al. [8] found that the CT radiomics signature can predict MNA of paediatric NB based on SVM high-precision, logistic and random forest classifiers, and having the SVM classifier the better prediction performance which is consistent with our study. The possible explanation is that SVM classifiers have more stable performance and are therefore more suitable for clinical promotion. Our previous studies [27] confirmed the value of 18F-FDGPET/CT in predicting MNA and 1p and 11q aberrations of paediatric NB, but it lacks radiological features, whereas the present study concluded by multifactorial logistic regression that necrosis is an independent risk factor of MNA, which has important clinical significance. It implies the presence of hypoxia within the intratumoural areas which is associated with the activation of a more aggressive phenotype, with a higher potential for metastatic spread and a poorer prognosis [26, 28]. In this study, the proposed C-R-R model showed good prediction performance in both the training cohort (AUC, 0.860) and validation cohort (AUC, 0.824). Furthermore, DCA confirmed the clinical utility of the C-R-R model.

Despite the significance of this study, we acknowledge there were some limitations. Firstly, this study adopted a single-centre design, and the sample size is still small. The proportion of patients with MYCN amplification was higher than that of the NB group in both the training cohort and the validation cohort, which may affect its diagnostic effectiveness. Therefore, the sample size should be increased in future studies. Secondly, our validation cohort used to test the model efficacy was from the same hospital as the training cohort, making it challenging to generalise our results to other hospitals. So, it is necessary to validate the results in a multi-centre cohort.

In conclusion, the C-R-R model is based on the radiomic signatures of 18F-FDG PET/CT, combined with clinical variables and radiological features. The model has good diagnostic performance and high accuracy in predicting MNA, which can provide useful image-based biological information for stratified treatment of patients.

### Supplementary Information

**Additional file 1: Table A1.** Delong tests’ results between different models.

## Data Availability

The datasets analysed during the current study are available from the corresponding author on reasonable request.

## Abbreviations

18F-FDG PET/CT: 18F-Fluorodeoxyglucose positron emission tomography/computer tomography
AUC: Area under the curve
HVA: Homovanillic acid
INRGSS: International Neuroblastoma Risk Group Staging System
LASSO: Least absolute shrinkage selection operator
LDH: Lactate dehydrogenase
MTV: Metabolic tumour volume
NB: Neuroblastoma
NSE: Neuron-specific enolase
ROC: Receiver operating characteristic curve
SF: Serum ferritin
SUVmax: Maximum standard uptake values
TLG: Total lesion glycolysis
VMA: Vanillylmandelic acid
VOI: Volume of interest
