# Supplementary material for: Predicting MYCN amplification in paediatric neuroblastoma: development and validation of a 18F-FDG PET/CT-based radiomics signature
Source: Insights Imaging. 2023 Nov 24;14:205. doi: 10.1186/s13244-023-01493-8 (PMC10673749; doi:10.1186/s13244-023-01493-8)
Supplement: Supplementary file 1 — Additional file 1: Table A1. Delong tests’ results between different models. [file 13244_2023_1493_MOESM1_ESM.doc]

**Predicting MYCN amplification in paediatric neuroblastoma: development and validation of a 18F-FDG PET/CT-based radiomics signature**

| **Table A1** Delong tests' results between different models | | | | | |
| --- | --- | --- | --- | --- | --- |
| **Training cohort** | LR | DT | SVM | C-R | C-R-R |
| LR | NA | 0.381 | 0.025* | 0.352 | < 0.001*** |
| DT |  | NA | 0.476 | 0.302 | < 0.001*** |
| SVM |  |  | NA | 0.412 | 0.005 ** |
| C-R |  |  |  | NA | < 0.001 *** |
| C-R-R |  |  |  |  | NA |
| **Validation cohort** | LR | DT | SVM | C-R | C-R-R |
| LR | NA | 0.921 | 0.852 | 0.035 | 0.154 |
| DT |  | NA | 0.847 | 0.946 | 0.474 |
| SVM |  |  | NA | 0.682 | 0.443 |
| C-R |  |  |  | NA | 0.007* |
| C-R-R |  |  |  |  | NA |
| **p* < 0.05; ***p* < 0.01; ****p* < 0.001 | | | | | |

**ELECTRONIC SUPPLEMENTARY MATERIAL**
